# Reliability of Sonography Measures of the Lumbar Multifidus and Transversus Abdominis during Static and Dynamic Activities in Subjects with Non-Specific Chronic Low Back Pain

**DOI:** 10.3390/diagnostics11040632

**Published:** 2021-04-01

**Authors:** Eleuterio A. Sánchez Romero, José Luis Alonso Pérez, Alberto Carlos Muñoz Fernández, Andrea Battaglino, Matteo Castaldo, Joshua A. Cleland, Jorge Hugo Villafañe

**Affiliations:** 1 Musculoskeletal Pain and Motor Control Research Group, Faculty of Health Sciences, Universidad Europea de Madrid, 28670 Madrid, Spain; albertocarlos.munoz@universidadeuropea.es; 2 Department of Physiotherapy, Faculty of Biomedical and Health Sciences, Universidad Europea de Madrid, Villaviciosa de Odón, 28670 Madrid, Spain; 3 Musculoskeletal Pain and Motor Control Research Group, Faculty of Health Sciences, Universidad Europea de Canarias, 38300 Tenerife, Spain; 4 IRCCS Fondazione Don Carlo Gnocchi, 20148 Milan, Italy; andrea.battaglino@edu.unito.it; 5 CNAP, Center for Sensory-Motor Interaction (SMI), Department of Health Science and Technology, Faculty of Medicine, Aalborg University, DK-9220 Aalborg, Denmark; matteo.castaldo@poliambulatoriofisiocenter.com; 6 Master in Sport Physiotherapy, University of Siena, 53100 Siena, Italy; 7 Department of Physical Therapy, Poliambulatorio Fisiocenter, 43044 Parma, Italy; 8 Department of Public Health and Community Medicine, Physical Therapy Program, Tufts University School of Medicine, Boston, MA 02111, USA; joshcleland@comcast.net

**Keywords:** low back pain, reliability, chronic pain

## Abstract

Purpose: The purpose of this study was to investigate the test-retest reliability of ultrasound (US) thickness measurements and the muscle contraction ratio (CR) of lumbar multifidus (LM) and transversus abdominis (TA) muscles in participants with and without nonspecific chronic low back pain (NCLBP). Methods: A total of 62 participants (37 with NCLBP, 25 without NCLBP) with participated in the study. The within-day and between-day reliability of US thickness measurements and CR in a lying (supine for TA and prone for LM) and sitting positions for both muscles (sitting on a gym ball with both feet on the ground or lifting one foot off the floor) were assessed. Reliability analysis was performed with intraclass correlations (ICCs) for these two static and dynamic positions. Results: Test-retest reliability was calculated to be good to high for the static position (ICC = 0.72–0.95) and the dynamic position (ICC = 0.74–0.94) sonographic measurements in both group of TA measurement. Test-retest reliability of LM measurements was good to high for the static position (ICC = 0.82–0.95) and the dynamic position (ICC = 0.85–0.97) sonographic measurements in both groups. Conclusions: US imaging is a highly reliable method for the assessment of TA and LM thickness muscles in the dynamic position in participants with and without NCLBP. The CR measures may be adequately reliable in assessing the function of the TA and LM muscles in participants with NCLBP and healthy ones.

## 1. Introduction

Chronic low back pain (CLBP) reportedly has a known cause (attributable to specific pathology, such as infections, tumors, osteoporosis, fractures, structural deformities, inflammatory pathology, radicular syndrome, etc.), inflammatory pathology, radicular syndrome or cauda equina) in only 10–15% of cases. Conversely, chronic low back pain of unknown cause (NCLBP) accounts for 85–90% of cases of chronic low back pain [1,2].

The probability of suffering acute low back pain at some point in life is close to 70–85%, and 90% of those will suffer more than one episode [3]. Of all the patients who suffer acute low back pain, 10–15% will suffer a chronification of their symptoms [1].

The etiology of NCLBP is related to peripheral and central sensitization processes [4]. Acute nociceptive pain is the result of noxious stimulation by damaged tissue. Damage tissue releases inflammatory molecules that favor the depolarization of the nociceptors and the transmission of the nociceptive impulse to higher centers, where the information is processed and interpreted as painful depending on the context and associated psychosocial factors [5].

If this peripheral nociceptive input is maintained over time in a repetitive manner, it sensitizes the nociceptors, decreases their activation threshold, and increases their excitability, causing hyperalgesia [6]. All of this contributes to a state of peripheral sensitization.

The persistent transmission of nociceptive signals from the periphery induces changes in the central nervous system. This can then provoke an expansion of the receptor fields of the nociceptive neurons, which favors the perception of pain in response to non-painful stimuli (allodynia) [7]. This state of hyperactivity and hyperexcitability at the medullary and cerebral levels is known as central sensitization.

The lumbar multifidus (LM) and transversus abdominis (TA) are deep stabilizing spinal muscles that are the most widely assessed in this context. Studies have reported that there is significant atrophy, asymmetry, reduced thickness, decreased cross-sectional area, and altered patterns of recruitments of the TA and LM muscles in individuals with LBP [8,9].

Ultrasound is a noninvasive and safe technique, since it does not use ionizing radiation. It is used for diagnosis and as a tool for the evaluation of pathological processes. It is also considered a valid and reliable tool for the evaluation of different variables, such as muscle cross-section area (CSA) and muscle strength [10]. It is an ideal method for the exploration of the musculoskeletal system, as there is the possibility to evaluate it both at rest and in movement, and it allows for an assessment of muscle morphology and function both qualitatively and quantitatively in real time. In addition, it can be used as a diagnostic method in clinical practice during the rehabilitation stages, being useful for both the professional and the patient, since it serves as biofeedback for both [10].

Romero et al. [10] studied the use of US to evaluate both muscle thickness and CSA and connective tissue associated with musculoskeletal features that may influence physical assessment.

In contrast, ultrasound showed that the CSA of the lumbar multifidus (LM) muscle was smaller on the side of the lumbar spine where pain was expressed, indicating muscle atrophy [11]. Using ultrasound, the study by Wallwork et al. [12] showed that patients with LBP had a significantly smaller LM CSA than healthy subjects.

O’Sullivan et al. [13,14] reported that the recruitment of spinal stabilizing muscles in the lumbo-pelvic region significantly increased during upright sitting compared to slouched or thoracic upright sitting. However, activity of the TA was not assessed.

Sonographic assessment of muscle thickness change closely reflects muscle activity at low levels (<40% maximum voluntary contraction) [15]. Thus, real-time ultrasound (US), as a valid and noninvasive method, can be a useful alternative to fine-wire electromyography for evaluating deep spinal muscle activity [16]. In addition, muscle contraction ratio (CR), as a potential indicator of muscle-tissue status (i.e., defined as contracted thickness/resting thickness of muscles), has recently been suggested for the estimation of muscle function [17]. To date, several reliability studies have assessed various aspects of LM and TA sonographic measurement during relatively static clinical tests in subjects with LBP [15,18,19]. However, there has been no study conducted to investigate the reliability of US measurements of LM and TA muscles thickness and CR during dynamic task in subjects with NCLBP.

Given that the use of ultrasound measurements is a new emerging science with potential to improve the diagnosis and treatment of patients with musculoskeletal pain, it seems that identifying acceptable reliability for any clinical measurement is essential in the selection of proper treatment. However, reliability of US measurements identified in static positions should not be generalized to dynamic positions, since the LM and TA muscle thicknesses are likely different between the two positions.

Therefore, the purpose of this study was to investigate the test-retest reliability of ultrasound (US) thickness measurements and the muscle contraction ratio (CR) of LM and TA muscles in participants with NCLBP during static and dynamic activities.

## 2. Materials and Methods

### 2.1. Design

The current study is a prospective observational cross-sectional study following the Strengthening the Reporting of Observational Studies in Epidemiology (STROBE) statement and checklist [20]. The study protocol was approved in January 2020 by the Ethical Committee of European University of Madrid (reference number CIPI/20/240). Informed consent was obtained from all participants and all procedures were conducted according to the Declaration of Helsinki.

### 2.2. Participants

A convenience sample of 62 subjects between the ages of 20 to 60 years old was recruited for the study from Universidad Europea de Madrid Department of Physical Therapy between September 2020 to December 2020. Due to the situation in Madrid caused by the SARS-COV-2 virus, the protocol used in the University Laboratory was the following:

1. Before entering to the center, shoes were disinfected by stepping on a mat soaked in hydrogen peroxide.
2. Then, the patient was given a temperature measurement with a digital thermometer, and hydroalcoholic gel was applied to the hands.
3. The use of surgical mask by the patient and FFP2 mask and face shield by the physiotherapist, in addition to a disposable gown, was mandatory.
4. Not having symptoms or having been in contact with people suspected of being with the current disease was verbally expressed.
5. During treatment, the stretcher, chair, ultrasound probe, and gloves were disinfected.
6. A safe distance was always maintained except during treatment.

Thirty-seven consecutive subjects, 47.2 ± 9.3 years of age, who had been diagnosed with NCLBP by a physician, were included in the study. The healthy control group consisted of 25 consecutive subjects, 43.3 ± 7.8 years old, from the same rehabilitation facility, as shown in Table 1.

The inclusion criteria for the NCLBP (experimental group) were localized back pain between the 12th rib and the gluteal folds lasting more than 3 months. A non-probabilistic sampling of judgmental or purposive sampling was performed. Subjects were excluded if they had history of pain radiating further than the buttock, sciatica, or other radicular involvement; spinal surgery; nerve root compression; neurological deficits; rheumatic diseases; diabetes; mental disorders; pregnancy; lower extremity injuries; or neuromuscular diseases [21].

The Visual Analogue Scale [22] was used to assess pain intensity and the Oswestry Disability Index (ODI) [23] to assess disability for all subjects with LBP. Additionally, all participants completed the Tegner Activity Rating Scale [24] to assess activity level.

### 2.3. Sonographic Assessment of the LM and TA

Sonography imaging in B-mode was performed using a SONOSCAPE E2 ultrasound machine and a 10.5–14 MHz linear probe with a 38 mm footprint by a single operator. In each subject, the LM and TA muscles thickness was imaged in a series of 2 postures: (1) Lying (static posture) at rest and contraction, and (2) sitting on gym ball (dynamic posture) at rest and contraction. In each position, 3 recordings were taken, and the average of the measures was use for the analysis.

**Figure 1:**
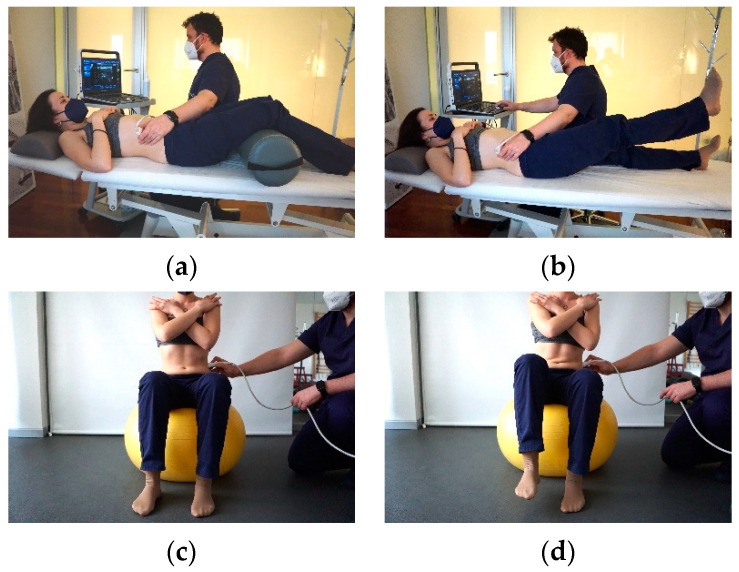
Images representing the positions used to measure TA muscle thickness at rest and contraction in different positions to obtain the CR (**a**) Transversus abdominis (TA) thickness measurement in supine lying (static at rest) with 60-degree hip flexion, (**b**) TA thickness measurement in supine lying during holding the lower extremity in the raised position, (**c**) TA thickness measurement in at rest on Fitball, (**d**) TA thickness measurement in contraction on Fitball with contralateral foot elevation.

The TA thickness measurement was obtained in 2 positions (static and dynamic) with 4 different tasks: (a) Supine lying (static at rest) with 60-degree hip flexion, a pillow under their head, and hands resting on the chest; (b) supine lying while holding the lower extremity in the raised position as Active Straight Leg Raise (ASLR) test (static at contraction), in which the participant was asked to raise the lower extremity 5 cm off the table without bending the knee [25]; (c) sitting comfortably on a gym ball (diameter: 65 cm) with a straight back, arms folded and resting on the opposite shoulders, and both feet on the floor (dynamic at rest); and (d) sitting on a gym ball, lifting the left foot off the floor by approximately 10 cm (dynamic at contraction) [26]. For the TA thickness measurement, a 50 mm, 5–7.5 MHz linear transducer head was placed along the line joining the anterior superior iliac spine (ASIS) to just below the ribcage in the mid-axillary line [27]. At the end of relaxed exhalation when the TA thickness was at its greatest, a clear image of the muscle thickness was captured and stored for later analysis [26]. To obtain the images and the corresponding TA measurements, a transverse plane approach was used at the point where the anterior superior iliac spine and the anterior axillary line intersect [27] In order to reliably reproduce the same ultrasound section, 2 perpendicular lines were marked so as to indicate the exact point where the transducer had to be placed, with an angulation of 90° with respect to the patient’s skin surface (Figure 1).

This methodology was performed in accordance with previous studies [12]. After applying the 10.5–14 MHz linear probe transducer head longitudinally and centrally on the target spinous process, the transducer was moved laterally to identify the relevant facet joint [28].

**Figure 2:**
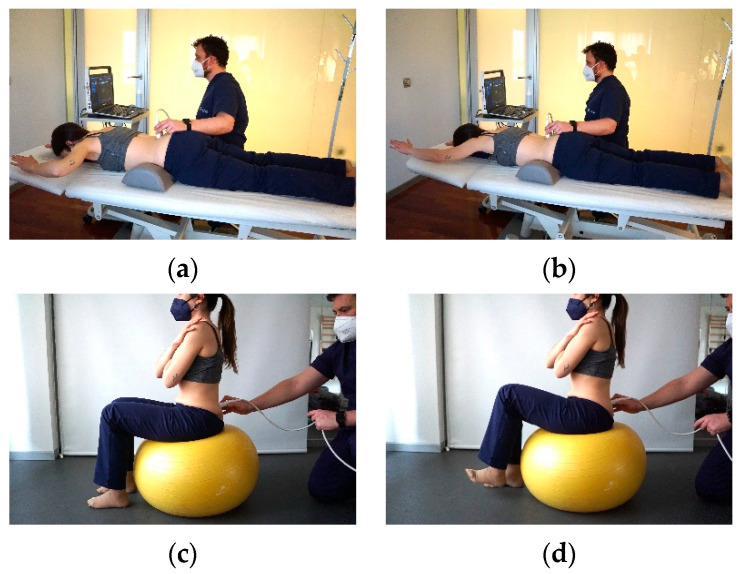
Images representing the positions used to measure LM muscle thickness at rest and contraction in different positions to obtain the CR (**a**) Lumbar multifidus (LM) thickness measurement in prone at rest, (**b**) LM thickness measurement in prone in contraction with arm elevation, (**c**) LM thickness measurement at rest on Fitball, (**d**) LM thickness measurement in contraction on itball with leg elevation.

The LM thickness measurement at the L4–L5 level was performed in 2 different positions (static and dynamic) with 4 different tasks: (a) Prone lying (static at rest) with a pillow under the abdomen to minimize lumbar lordosis [15,29]; (b) prone lying during the contralateral arm lift task (CLAT) (static at contraction), where the subject was instructed to “lift your upper approximately 5 cm off the table” while her or his upper limbs were repositioned overhead, elbows flexed to 90 degrees, and shoulders abducted to 120 degrees as measured using a goniometer [15]; (c) sitting on the gym ball with both feet on the floor (dynamic at rest); and (d) sitting on the gym ball with lifting the left foot off the floor (dynamic at contraction). The details of the last 2 positions (c and d) are the same as the last 2 positions of TA thickness measurement described above. To obtain the images and the corresponding measurements in a reliable and reproducible manner in the different tasks of the study, we located the spinous process of L5 ascending cranially from the sacrum. From L5, we also positioned the spinous process of L4 cranially. Both spinous processes were marked with an indelible marker. Once both spinous processes were marked, a line was also marked at the intermediate point between the spinous process of L4 and L5, which coincided with the position where the ultrasound transducer was placed to obtain a cross-sectional image of the multifidus musculature and thus be able to reliably reproduce the different measurements to be taken in the study. The angle at which the transducer was placed was always at an angulation of approximately 90° with respect to the patient’s skin, both in passive and dynamic measurements [15,29] (Figure 2).

### 2.4. Image Analysis

**Figure 3:**
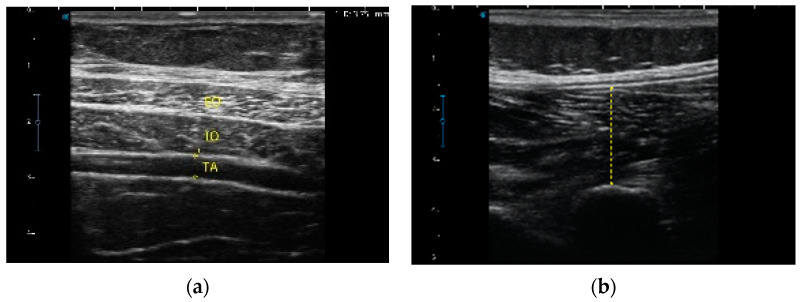
(**a**) TA thickness measurement following a transverse section of the abdominal wall for observation of the External Oblique, Internal Oblique and TA. (**b**) LM thickness measurement following a longitudinal section of the paraspinal musculature between L4-L5 for observation of the LM.

All sonographic images were processed offline using software (Image J; US National Institutes of Health, Bethesda, MD, USA). The cursor points carefully measured the TA and LM muscle thickness. The transducer was not moved during the testing procedure. Linear measurements between the superficial and deep hyperechoic fasciae perpendicular to the muscle fibers in millimeter (mm) were taken as the TA muscle thickness. The thickness of the LM was taken as the linear distance between the most posterior portion of the target facet joint and the thoracolumbar fascia. To ensure standardized placement of the measurement line, a straight vertical line through the center of the sonographic image was used. Contraction thickness ratio of TA and LM muscles were calculated as (CONTRACTION)-(REST)/REST [29,30] (Figure 3).

### 2.5. Procedure

The study protocol was the same for experimental and control groups. All examinations were performed in a quiet and draught-free laboratory. All participants (with and without NCLBP) were evaluated on 3 separate sessions. The first session was performed on day 1 then 1 h later to determine within-day reliability. After 3 days, the third session was completed for the between-day reliability assessment. The subjects testing positions and the order of measurements were randomly selected to avoid an order effect. All measurements and data processing (i.e., US measurements) were performed by the trained physical therapist who had a European Master’s Degree in ultrasound techniques and a postgraduate professor in ultrasound techniques.

### 2.6. Statistical Analysis

Data were analyzed using SPSS version 25.0 (SPSS Inc., Chicago, IL, USA). Intrarater reliability for the average of 3 measures in muscle thickness and contraction thickness ratio of TA and LM muscles was assessed by the intraclass correlation coefficient (intraclass correlation (ICC) 3.1; method: Alpha, 2-way mixed, consistency). We made use of model 3 (ICC 3.1) because only 1 rater assessed the same population. The ICCs were classified as follows: <0.70, poor correlation; 0.70–0.79, fair correlation; 0.80–0.89, good correlation; 0.90–1.00, high correlation. The standard error of measurement (SEM = pooled SD √1—ICC) and the minimal detectable change (MDC) for a 95% confidence interval (MDC = SEM × z × √2) were calculated. The MDC reflects the minimal detectable change in a score that, with *p* < 0.05, can be interpreted as the relevance of any changes recorded after an intervention. The SEM reflects the error of the instrument itself.

## 3. Results

### 3.1. Demographic and Clinical Data of Participants

In the current study, 62 participants (46.3 ± 8.2 years old) with complete set of valid and immediate re-test results (less than 1 week) participated. Patient with LBP had mild to moderate levels of pain and disability (Table 1).

### 3.2. Variation of the US and CR Parameters

The resting and contracted thickness of TA for the control group ranged from 3.8 mm to 4.4 mm and from 4.7 mm to 5.3 mm in both positions, respectively, and the resting and contracted thickness of TA for experimental group ranging from 4.1 mm to 4.7 mm and from 4.2 mm to 5.1 mm in both positions, respectively. The resting and contracted thickness of LM for the control group ranged from 31.0 mm to 32.1 mm and from 37.4 mm to 39.3 mm in both positions, respectively, and the resting and contracted thickness of LM for the LBP group ranged from 31.3 mm to 33.9 mm and from 37.5 mm to 41.2 mm in both positions, respectively. The smallest and highest CR of the TA and LM muscles belonged to patients in the experimental group in the dynamic position and a subject in the control group in the dynamic position respectively.

### 3.3. Test-Retest Reliability

The sonographic measurements of the TA in “static at contraction” position for within-day reliability was good for the experimental group (ICC = 0.88) and high for the control group (ICC = 0.96). Differently, the between-day reliability was poor (ICC = 0.72) for the experimental group and high (ICC = 0.95) for the control group. For the “dynamic at contraction” position, the within-day reliability of the sonographic measurements of the TA was high for both groups (ICC = 0.90 in the experimental group, ICC = 0.97 in the control group), whereas the between-day reliability was good for the experimental group (ICC = 0.85) and high for the control group (ICC = 0.95).

The within-day reliability of the sonographic measurements of the LM for “static at contraction” position was high for both groups (ICC = 0.94 in experimental group; ICC = 0.92 in control group), whereas the between-day reliability was good (ICC = 0.82) for the experimental group and still high (ICC = 0.94) for the control group. In the “dynamic at contraction” position, the experimental group presented high (ICC = 0.90) and good (ICC = 0.85) within-day and between-day reliability, respectively, and the control group presented high reliability for both parameters (ICC = 0.93 and ICC = 0.93).

In within-day and between-day reliability, the SEM of the sonographic measurements in the “static at contraction” position of the TA in the experimental group ranged from 0.54 to 0.94, respectively. In the control group, the SEM of within-day and between-day reliability in the same position varied to a small extent, from 0.46 to 0.52, respectively. In relation to the “dynamic at contraction” position, for the within-day and between-day reliability, the SEM of the sonographic measurements of the TA ranged from 0.56 to 0.75, respectively, for the experimental group and from 0.33 to 0.20, respectively, for the control group. Overall, we had higher SEM in the experimental group compared with participants in the control group.

For within-day and between-day reliability, the SEM of TA CR in the experimental group ranged from 0.08 to 0.11, respectively, in the static position and remained at 0.15 in the dynamic position. In the control group, the same parameter ranged from 0.13 to 0.1, 6 respectively, in the static position and from 0.07 to 0.08 in the dynamic position. For within-day and between-day reliability, the SEM of LM CR in the experimental group ranged from 0.06 to 0.03, respectively, in the static position and remained at 0.04 in the dynamic position. In the control group, the same parameter ranged from 0.06 to 0.05, respectively, in the static position and from 0.10 to 0.07 in the dynamic position. In total, the SEM of TA CR was higher than the SEM of LM CR in both groups.

Likewise, MDC trends in within-day and between-day reliability for TA in the experimental group ranged from 0.20 to 0.31, respectively, in the static position and from 0.16 to 0.14, respectively, in the dynamic position. In the control group, these values ranged from 0.35 to 0.45, respectively, in the static position and from 0.15 to 0.22, respectively, in the dynamic position. This value for LM in within-day and between-day reliability in the experimental group remained 0.11 in the static position and ranged from 0.16 to 0.14, respectively, in the dynamic position, whereas it ranged from 1.09 to 0.15, respectively, in the static position and from 0.23 to 0.25, respectively, in the dynamic position. Overall, we had higher MDC in experimental group compared with controls, as shown in Table 2.

## 4. Discussion

The results of this study suggest that US measurements of muscle thickness and CR in participants with and without NCLBP during static and dynamic positions exhibit good to high reliability. These results are consistent with the available literature on US measurements of TA and LM muscles thickness [31,32].

Ainscough-Potts et al. [26] assessed the intrarater reliability of US to measure TA thickness of healthy subjects in relaxed sitting on a chair compared to sitting on a gym ball, and high reliability (ICC: 0.97–0.99) was reported. Arab et al. [31] found high ICC (0.85–0.95) and low SEM (0.19–0.78 mm) and MDC (0.52–2.15 mm) for the TA muscle during lying and sitting on the gym ball in individuals with and without LBP, respectively.

Also, small SEM and MDC scores were obtained in the current study, which is similar to the results found by Arab et al. [31]. We found higher SEM and MDC in the LBP group compared with participants without LBP. Additionally, the SEM and MDC of LBP group were higher in the dynamic position than the static position.

Higher SEM and MDC in the NCLBP group in the current study may be attributed to the variability of motor control or muscle recruitment patterns in the patients [33]. Large variability of the muscles thickness may result from the multiple modulation strategies of the mentioned muscles. Therefore, the use of multiple modulation strategies could have a direct effect on the average of the three trials, thereby increasing the variance in the measure.

It has been previously shown LBP patients have differences in muscles compared to healthy subjects. LBP patients showed reduced capacity of rapid muscle activation [34], higher fatty infiltrations [35], muscle atrophy [36], changes in muscle recruitment time [37], and the reduction of trunk muscle strength and endurance [38,39]. These findings certainly affect the patient’s motor control of posture and movement.

It is known that when using between-day intraexaminer MDCs, if a patient with low back pain initially shows a change in LM muscle thickness of 10%, after rehabilitation, it would need to increase to at least 21% for the examiner to obtain 95% confidence that a true change has occurred [40]. For the TA muscle, the required changes are even greater.

The dynamic position (sitting on gym ball) is harder than the static position (lying), and participants without LBP can keep the dynamic position easier with less displacements and variability during these tasks than subjects with LBP.

Reliability of US measurements of muscle thickness can be impacted by several factors and sources of error, including the participants, operator and testing position, accuracy of marking the fascial bands, position of subject or transducer, and landmark detection [31].

Although changes in the thickness of these muscles are an excellent measure for assessing function over time, it should be noted that these measurements incorporate errors associated with the measurements when comparing those taken during rest and contraction [19].

When extrapolating these results to clinical practice, the influence of the examiner’s ability to give instructions, the patient’s state of mind, and the patient’s previous experience in motor control training must be taken into account.

The good to high reliability of US measurements in the current study considering the evaluation of/in the dynamic position may be due to several reasons, i.e., clear image was frozen on the screen, the muscle thickness was measured carefully by cursor points, and the US transducer was not displaced during the testing procedure [15].

These findings may help clinicians in the US assessment of muscle thickness and CR in LBP patients, as from our results changes depending on the position of the patients. In the task required, the changes in results do not influence the reliability of US assessment.

Therefore, US assessment may be a reliable tool to help clinicians in monitoring TA and LM function and changes during the course of intervention.

Further, future studies may be able to tell us if CR and thickness change can be linked to clinical improvement in LBP patients.

In fact, in future studies on NCLBP treatment, the assessment of CR and thickness may be used as a reliable tool for both baseline assessment and functional improvements and may therefore help to develop more effective interventions.

We believe that the high consistency of CR (based on ICC values) is the major finding of this work, and it is more consistent for the NCLBP group. Given that the scientific literature supports the use of strength training to improve the ability to develop an effective contraction [41] and improve the symptomatology of chronically painful muscles [42], we believe that the CR is an excellent parameter not only for the assessment but also for the evolution of the patient in rehabilitation.

In this view, our findings encourage the use of US measurement in the clinical setting as a reliable tool also in NCLBP, regardless their different variability of muscle recruitment and the different positions of assessment.

### Limitations of the Study

Several limitations exist within the current study. We did not assess interrater reliability. Also, the muscle thickness and CR of other trunk muscles such as the oblique muscles and LM muscles of other levels of lumbar vertebra were not evaluated. The results might not be extrapolated to other levels. More studies are recommended to collectively perform this study in other abdominal and LM muscles. It is suggested to assess the within-day and between-day reliability of US measurements of these muscles in subjects with and without NCLBP with other postures during different dynamic positions.

## 5. Conclusions

The US measurements of TA and LM thickness and CR showed good to excellent within-day and between-day reliability and low SEMs and MDCs during the lying and sitting positions on the gym ball in subjects with and without NCLBP when taken by a single examiner. The CR measures may be adequately reliable and helpful to assess the function of the TA and LM muscles in participants with NCLBP and healthy ones.

Taking into account the high consistency of CR, we believe that the CR is an excellent parameter not only for the assessment but also for the evolution of the patient in rehabilitation.

US assessment may be therefore a useful instrument to assess and design interventions for NCLBP patients.

## Figures and Tables

**Table 1:** Demographic data of the participants in each group (Mean ± SD).

Variable	Experimental Group (*n* = 37)	Control Group (*n* = 25)	*p*-Value
Age, years	47.2 ± 9.3	43.3 ± 7.8	0.04
BMI, kg/m^2^	25.6 ± 3.4	24.8 ± 3.6	0.02
VAS	3.6 ± 1.4	-	-
ODI	22.6 ± 12.1	-	-
Tegner scale	3.4 ± 0.7	3.1 ± 0.4	0.05

SD: Standard deviation, BMI: Body mass index, VAS: Visual Analogue Scale, ODI: Oswestry Disability Questionnaire.

**Table 2:** SD, ICC, SEM and MDC values for within-day and between-days reliability of the ultrasound thickness measurements (in mm) and contraction ratio of the TA and LM muscles during different testing positions in both groups.

Position		Muscle Task	Experimental Group								Control Group							
			Within-Day				Between-Days				Within-Day				Between-Days			
			SD	ICC	SEM	MDC	SD	ICC	SEM	MDC	SD	ICC	SEM	MDC	SD	ICC	SEM	MDC
Static	**TA**	**Rest**	1.50	0.93	0.40	1.21	1.70	0.92	0.46	1.28	3.11	0.94	0.55	1.39	2.43	0.90	0.69	1.91
		**Contraction**	1.81	0.88	0.54	1.45	1.81	0.72	0.94	2.21	2.63	0.96	0.46	1.27	2.52	0.95	0.52	1.44
		**Contraction ratio**	0.4	0.95	0.08	0.20	0.40	0.93	0.11	0.31	0.29	0.86	0.13	0.35	0.42	0.80	0.16	0.45
	**LM**	**Rest**	5.27	0.91	1.48	4.23	5.26	0.82	2.31	6.28	6.58	0.98	0.50	1.63	7.01	0.98	0.44	1.79
		**Contraction**	6.37	0.94	1.68	4.81	6.42	0.82	2.55	7.30	9.60	0.92	2.38	7.07	9.98	0.94	2.30	6.31
		**Contraction ratio**	0.19	0.95	0.06	0.11	0.11	0.91	0.03	0.11	0.21	0.90	0.06	1.09	0.23	0.93	0.05	0.15
Dynamic	**TA**	**Rest**	1.26	0.90	0.40	0.98	1.46	0.74	0.69	2.07	1.55	0.93	0.40	1.10	1.38	0.94	0.31	0.88
		**Contraction**	1.87	0.90	0.55	1.63	2.10	0.85	0.75	2.16	1.68	0.97	0.33	0.70	1.59	0.95	0.20	0.92
		**Contraction ratio**	0.39	0.93	0.15	0.31	0.51	0.94	0.15	0.31	0.35	0.95	0.07	0.15	0.35	0.93	0.08	0.221
	**LM**	**Rest**	7.92	0.96	1.42	4.05	7.88	0.93	2.10	5.95	9.42	0.95	1.78	5.08	9.21	0.97	1.88	5.19
		**Contraction**	8.32	0.90	2.60	7.10	8.57	0.85	3.40	8.91	11.05	0.93	2.70	7.48	10.95	0.93	2.79	7.79
		**Contraction ratio**	0.27	0.97	0.04	0.16	0.27	0.96	0.04	0.14	0.41	0.93	0.10	0.23	0.41	0.93	0.07	0.25

SD: Standard deviation, LBP: Low back pain, TA: Transversus abdominis, LM: Lumbar Multifidus, ICC: Intraclass correlation coefficient, SEM: Standard error of measurement, MDC: Minimal detectable change. All SEM and MDC values are in millimeter.

## Data Availability

Data available on request due to privacy and ethical restrictions.
